# Enhanced sunlight-driven photocatalytic property of Mg-doped ZnO nanocomposites with three-dimensional graphene oxide/MoS_2_ nanosheet composites

**DOI:** 10.1039/c8ra02382d

**Published:** 2018-05-14

**Authors:** Chuansheng Chen, Wei Mei, Weiwei Yu, Xi'an Chen, Longhui Zeng, Yuenhong Tsang, Zisheng Chao, Xiaoyan Liu

**Affiliations:** College of Materials Science and Engineering, Changsha University of Science and Technology Changsha 410114 China jxccs1934@csust.edu.cn +86-731-85258490; Zhejiang Key Laboratory of Carbon Materials, College of Chemistry and Materials Engineering, Wenzhou University Wenzhou 325027 People's Republic of China chenxa1981@163.com; Department of Applied Physics, Hong Kong Polytechnic University Hong Kong 999077 China

## Abstract

Graphene oxide (GO) has been the focus of attention as it can enhance the photocatalytic activity of semiconductors due to its large specific surface area and remarkable optical and electronic properties. However, the enhancing effect is not ideal because of its easy self-agglomeration and low electronic conductivity. To improve the enhancing effect of GO for ZnO, three-dimensional GO/MoS_2_ composite carriers (3D GOM) were prepared by electrostatic interactions and then, Mg-doped ZnO nanoparticles (MZ) were supported on the surface of 3D GOM by utilizing the layer-by-layer assembly method. Compared with GO/Mg-ZnO composite (GOMZ), the resultant three-dimensional GO/MoS_2_/Mg-ZnO composite (GOMMZ) exhibited excellent photocatalytic performance due to the effective synergistic effect between GO and MoS_2_ sheet, and its degradation rate was nearly 100% within 120 min of exposure to visible light; this degradation rate was nearly 8 times higher than that of the GOMZ composite. Moreover, the introduction of the MoS_2_ sheet intensified the photocurrent density of the GOMZ composite and endowed it with optical memory ability.

## Abstract 2

Graphene oxide (GO) has been the focus of attention as it can enhance the photocatalytic activity of semiconductors due to its large specific surface area and remarkable optical and electronic properties.

## Graphical abstract

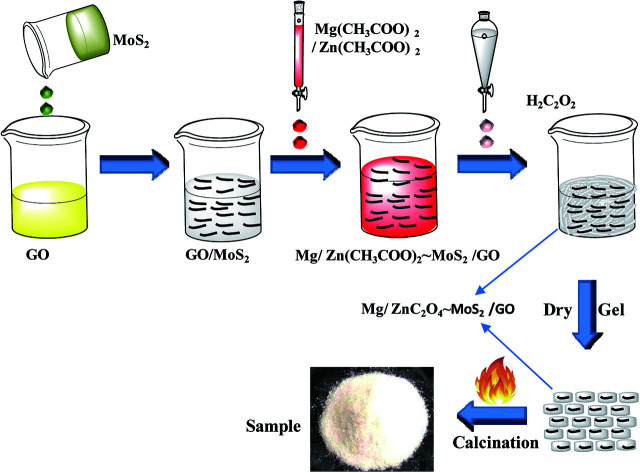

## 1 Introduction

ZnO nanostructures are one of most promising catalysts due to their superior properties, low cost, abundance, non-toxicity, and high surface activity. However, pure ZnO nanostructures still suffer from high recombination rate of photo-induced electron–holes pairs, low utilization of sunlight and low conversion efficiency of light.^1–3^ Until now, many attempts have been made to improve the photocatalytic activity of these structures such as doping, metal nanoparticle depositing, co-catalyst or sensitizer modification, copolymerization, catalyst carrier and semiconductor combination.^4–10^ Because of the similar ionic radii of Zn and Mg, Mg-doping can significantly enhance the photocatalytic activity of ZnO nanostructures by forming oxygen or zinc vacancies.^11^ However, Mg-doped ZnO nanostructures still possess very high recombination rate of photo-induced electrons.

Graphene has attracted ample attention as a photocatalyst composite, which can rapidly capture and transfer photo-induced electrons, owing to its large specific surface area, strong electronic storage ability, and high carrier mobility.^12,13^ Because graphene can transfer photoexcited electrons faster and hinder the aggregation of semiconductor nanostructures, loading semiconductors on a graphene sheet can enhance photocatalytic activity significantly. It has been reported that graphene can further enhance the photocatalytic activity of Mg-doped ZnO composites in practical applications.^14,15^ However, it is very difficult for graphene sheets to load semiconductors due to the poor compatibility, severe self-restacking and self-agglomeration.

MoS_2_ is a p-type semiconductor, and it shows the strongest photo-response in the visible region due to its narrow band gap energy and a potential sensitizer.^16^ Until now, a number of MoS_2_-based heterojunctions (MoS_2_/ZnO, MoS_2_/TiO_2_, *etc.*) have been reported for photocatalytic reactions, and the results show that MoS_2_-based heterojunctions exhibit enhanced photocatalytic activity under visible light.^17–22^ Recently, significant attention has been devoted to apply the two-dimensional layered MoS_2_ sheet for improving the photocatalytic activity owing to the unique nature of its open band gap, distinctive physicochemical properties, native vacancy defects and low electrical conductivity.^23–25^ Unfortunately, the photocatalytic activity of pure layered MoS_2_ sheet is very weak due to quick recombination of excitons, which impedes charge transfer at the surface.

Construction of three-dimensional (3D) composites with graphene and nanostructures has aroused great attention because of the strong photocatalytic activity caused by the effective synergistic effect.^26–29^ Constructing 3D graphene/MoS_2_ sheets can alleviate the agglomeration of graphene because of the space resistance effect of MoS_2_, and it can also quickly transfer photoelectrons by the “synergistic effect” between graphene and MoS_2_ sheets.^30–34^ Experimental results demonstrate that constructing 3D composites by using graphene and MoS_2_ sheets can improve the photocatalytic activity and electrocatalytic performance.^35–40^

Herein, a 3D graphene oxide/MoS_2_ nanoplate catalyzer carrier was constructed by electrostatic attraction and then, a 3D GOMMZ composite was obtained by a facile constant-temperature coprecipitation method. The photocatalytic activity and photo-electrochemical performance of the resultant GOMMZ composite were studied in detail. Furthermore, the assembly and photocatalytic mechanism of the 3D GOMMZ composite were discussed.

## 2 Experimental

### 2.1 Preparation of GOMMZ hybrids

Graphene oxide was prepared by a modified Hummers methods, and the concentration of the obtained GO was 0.8 g L^−1^. A well-dispersed MoS_2_ nanoplate solution was prepared by the mechanical shear exfoliation method using cetyltrimethyl ammonium bromide (CTAB), according to our previous report.^41^ In a typical procedure, 10 g MoS_2_ and 0.5 g CTAB were mixed in 500 mL deionized water under sonication and stirring for 120 min. An L5M high shear laboratory mixer (made by Silverson Machines Ltd. UK.) driven by a 250 W motor at a speed of 7000 rpm was put under the mixture solution for 3 h. Afterwards, the suspension of MoS_2_ nanoplates was sonicated for 3 h to produce MoS_2_ nanoplate suspension, and its concentration was about 1.0 g L^−1^.

**Fig. 1:**
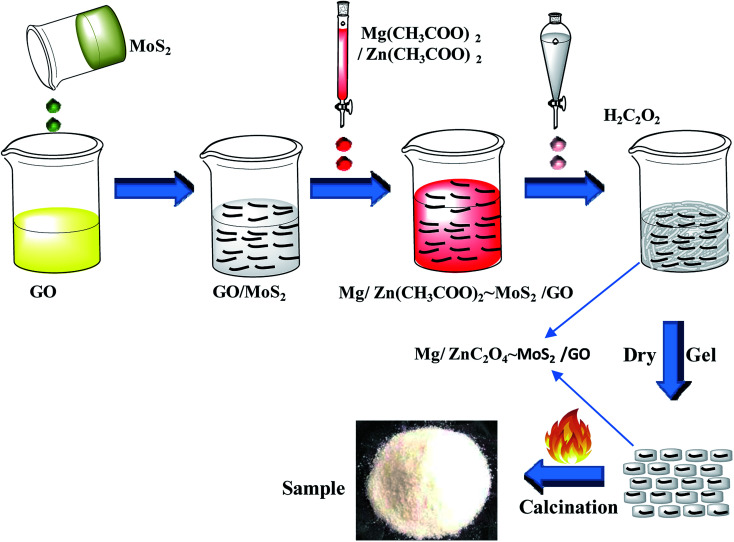
The synthetic procedure of GOMMZ composite.

A GOMMZ hybrid was prepared according to the following steps: (1) GO solution (0.012 g, 15 mL) and 2 mL MoS_2_ nanoplate solution (0.002 g) were mixed, forming the GO/MoS_2_ solution. (2) ZnC_2_O_4_·2H_2_O (5.487 g) and 0.604 g MgC_2_O_4_·2H_2_O were dissolved 20 mL deionized water and then, the mixed solution was added into the GO/MoS_2_ solution. (3) A certain amount of oxalic acid solution (0.25 mol L^−1^) was added to the mixed solution of GO/MoS_2_ dropwise in a thermostatic water bath maintained at 80 °C. (4) After drying the solution, the precursor was annealed at 500 °C for 2 h under a nitrogen gas atmosphere. The final products were recorded as GOMMZ, in which the mass fractions of graphene, Mg–ZnO and MoS_2_ were 0.56 wt%, 0.09 wt%, and 99.35 wt%, respectively. The whole process of the synthesis of GOMMZ composite is shown in Fig. 1. For comparison, GO/Mg-doped ZnO and MoS_2_/Mg-doped ZnO composites prepared under the same conditions were named as GOMZ (the mass fractions of graphene and Mg–ZnO were 0.56 wt% and 99.44 wt%, respectively) and MMZ, respectively.

### 2.2 Characterization

The X-ray diffraction (XRD) studies were performed on a Philips PW 1710 diffractometer with Cu Kα_1_ radiation. Scanning electron microscopy (SEM) studies were carried out with on a S-4800 field emission scanning electron microscope. Transmission electron microscopy (TEM) was conducted on a JEM-3010 transmission electron microscope. Raman spectroscopy was executed by Renishaw inVia. Brunauer–Emmett–Teller (BET) surface areas were determined using a nitrogen adsorption analyzer (ASAP2020HD88). Fluorescence measurements and quantum yields were characterized on a Hitachi F4500 fluorescence spectrophotometer, and the wavelength of excitation was 340 nm. UV-vis diffuse reflection spectra were recorded on a TU-2550 spectrophotometer. The Zeta potentials of diluted suspensions (0.01 wt%) were measured with a Zetaplus analyzer (90 plus particle size analyzer, Brookhaven, NY, USA). Each sample was ultrasonicated for 1 h prior to analysis.

RhB was used as a model dye to evaluate the photocatalytic activity of GOMMZ composites. The typical photocatalytic experiments were mentioned in our previous reports.^15,42^ Briefly, 150 mg samples were dispersed in 300 mL of 1 × 10^−5^ mol L^−1^ RhB, which was magnetically stirred for 20 min without interruption to ensure full suspension of particles before light illumination. Next, the photocatalysis solution was irradiated under 500 W Xe lamp. Then, 5 mL solution was extracted and subsequently centrifuged at 4000 rpm for 10 min. The absorbance of the solution was measured by a TU-2550 spectrophotometer at the maximum absorption wavelength of RhB (554 nm). The degradation ratio was calculated with *C*/*C*_0_, where *C*_0_ is the original concentration and *C* is the residual concentration at different times.

The experiment of photocatalytic memory was as follows: the resultant GOMMZ composites (150 mg) were dispersed in 300 mL deionized water and then, they were exposed to ultraviolet light irradiation for 6 h. After the light was removed, 1 mL RhB solution (5.0 × 10^−4^ mol L^−1^) was added to the above-mentioned solution. For comparison, GOMZ composites were irradiated under the same conditions.

Cyclic voltammetry (CV) and AC impedance (EIS) spectra were obtained using the CHI 660 electrochemical workstation. The electrolyte used for the CV test was 0.5 mol L^−1^ diluted sulfuric acid solution. The electrolyte used for the EIS test and photocurrent spectrum was 1 mol L^−1^ sodium sulfate solution. The working electrode of CV and EIS tests was a glassy carbon electrode coated with 10 μL mixed liquor, which was composed of 5 mg sample, 950 μL anhydrous ethanol and 50 μL Nafion. The working electrode used for obtaining the photocurrent spectrum was an FTO glass electrode, and it was prepared as follows: initially, 20 mg polyethyleneglycol (molecular weight, 1000) was dissolved in 50 mL absolute alcohol. Subsequently, 20 mg of resultant samples was added into the polyethyleneglycol solution under sonication and stirring for 60 min. Then, the sample solutions were brushed on the FTO glass (1 cm × 1 cm). Finally, the FTO glass was dried at 80 °C.

## 3 Results and discussion

**Fig. 2:**
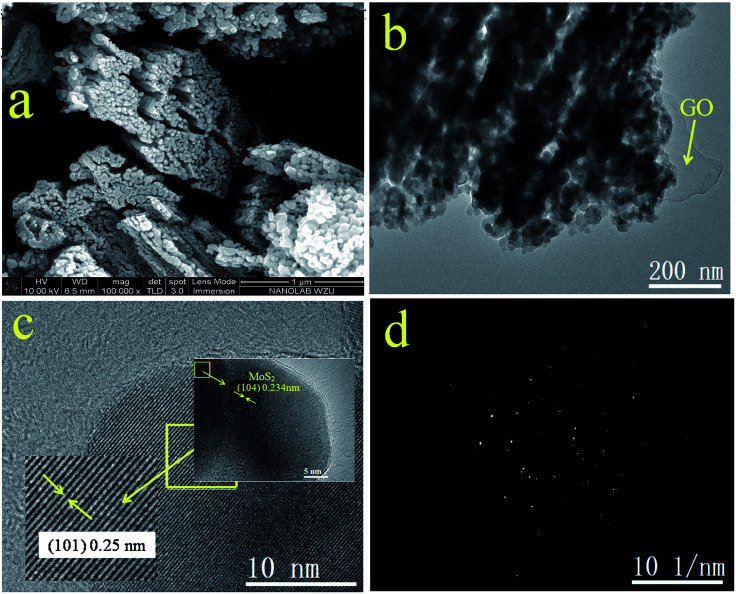
SEM (a), LRTEM (b), and HRTEM images (c) and SAED patterns (d) of GOMMZ sample.

The morphology of GOMMZ samples was studied using SEM images, TEM images and SAED patterns, as shown in Fig. 2. It was very clear that the GOMMZ composites were flat columnar with a large number of slits through holes (Fig. 2a), and they were composed of many small nanoparticles loaded on GO sheets (shown in Fig. 2b). The HRTEM image (Fig. 2c) demonstrated that the Mg-doped ZnO nanoparticles showed clear fringes, and the atomic interspacing was about 0.25 nm corresponding to the (101) plane of a ZnO crystal; the atomic interspacing of MoS_2_ was about 0.234 nm corresponding to the (104) plane. The homologous SAED pattern (Fig. 2d) indicated that polycrystal rings consisted of many homogeneous and tiny single-crystals, which implied that the ZnO nanoparticles had a high crystallization degree.

**Fig. 3:**
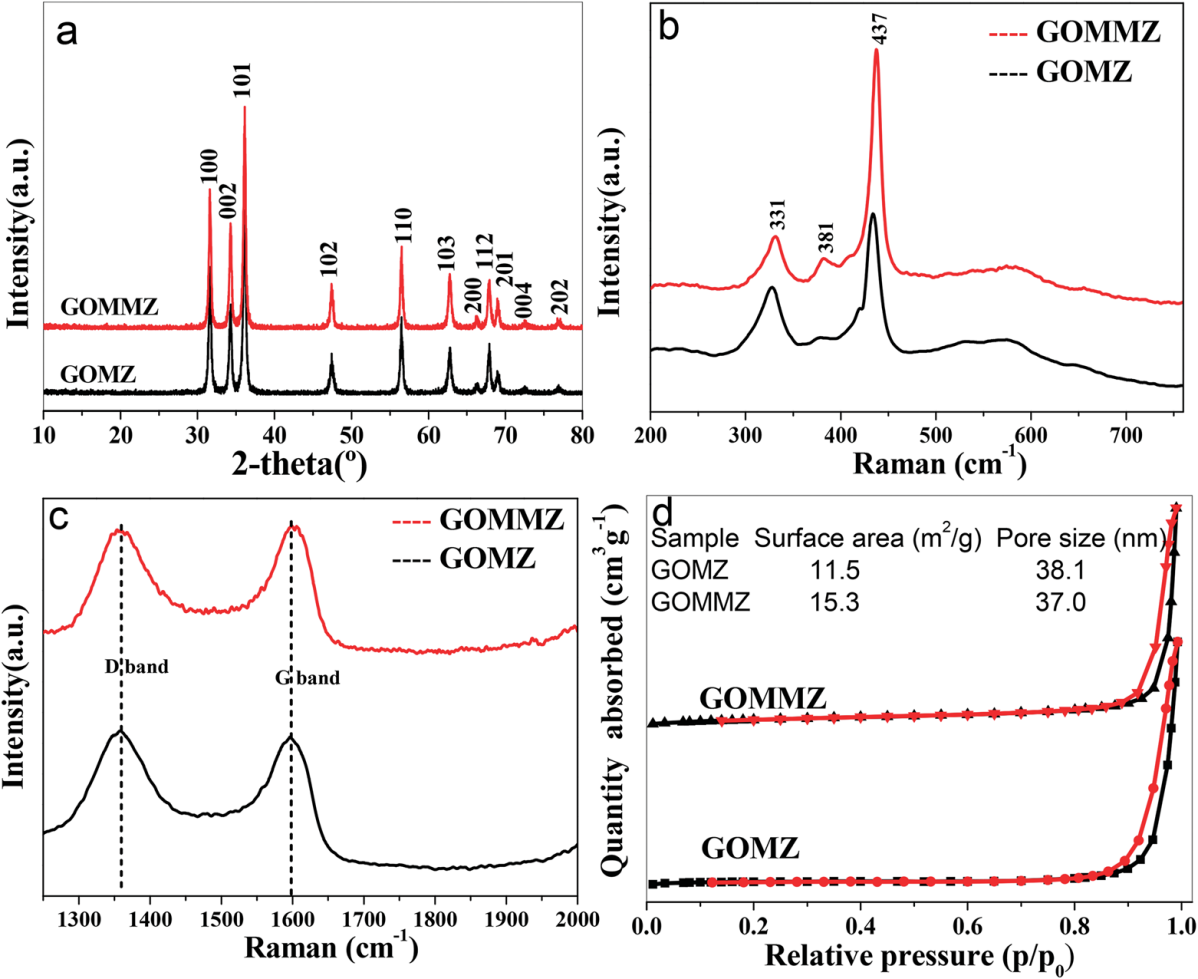
(a) XRD patterns, (b) and (c) Raman spectra of different samples, (d) the surface area, pore size and nitrogen adsorption–desorption isotherms of different samples.

To verify the composition and crystallinity of the resultant GOMMZ and GOMZ samples, the samples were tested with an X-ray diffractometer (XRD). Fig. 3a shows the XRD patterns of GOMMZ and GOMZ. The diffraction peaks of the two samples could be indexed as hexagonal wurtzite ZnO (JCPDS no. 36-1451), and the crystallinity of GOMMZ was higher than that of GOMZ. No other peaks of GO, carbon and MoS_2_ were observed, which may be ascribed to their low contents. Moreover, the absence of the peaks of magnesium oxide suggested that magnesium was doped in the lattice of ZnO. The structures of GOMMZ and GOMZ composites were verified by Raman spectra, as shown in Fig. 3b. Four vibration peaks were detected from 300 cm^−1^ to 600 cm^−1^ in both the samples; these observations were consistent with our previously reported results.^33^ In detail, the vibration at 331 cm^−1^ could be assigned to the second-order of Raman scattering arising from zone-boundary phonons 2-E_2_ (M) of ZnO. The peaks at 381 cm^−1^ and 437 cm^−1^ were assigned to A_1_ transverse optical (TO) and the E_2_ (high) mode, arising from the oscillation of Zn–O, which indicated that the resultant ZnO was in the wurtzite phase. Moreover, the peak at 574 cm^−1^ was assigned to A_1_ longitudinal optical mode (LO) of ZnO. In addition, the D and G modes of GO were observed at about 1335 cm^−1^ and 1601 cm^−1^, respectively. The peak at 1335 cm^−1^ was assigned to the zone center phonons of E_2g_ symmetry and sp^2^ hybridized carbons, and the peak at 1601 cm^−1^ was assigned to local defects and instability of the carbon structure of GO. By comparing the intensities of G and D modes, we found that the *I*_G_/*I*_D_ value of GOMMZ (0.99) was higher than that of GOMZ (0.92), which indicated that the reduction degree of GO in GOMMZ was higher than that in GOMZ. Fig. 3d shows the surface area, pore size and nitrogen adsorption–desorption isotherms of GOMMZ and GOMZ samples. The curves belonged to type IV isotherms with H3-type hysteresis loops, which indicated that the GOMMZ and GOMZ samples had slit-shaped pores, and the pore size was continuous distribution from mesoporous and macroporous. Furthermore, the introduction of MoS_2_ increased the surface area of the GOMMZ sample, whereas it slightly decreased the pore size of the GOMMZ composite.

**Fig. 4:**
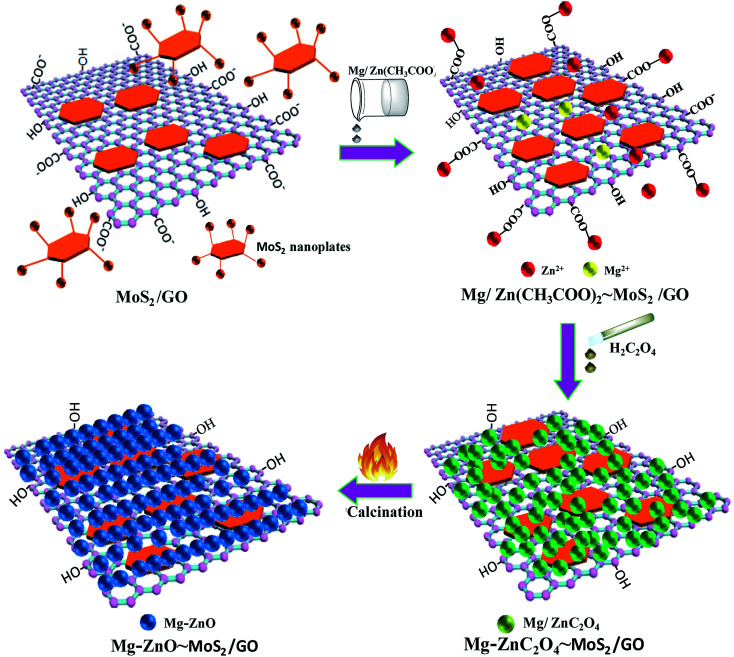
Schematic diagrams for the fabrication of GOMMZ composites.

**Fig. 5:**
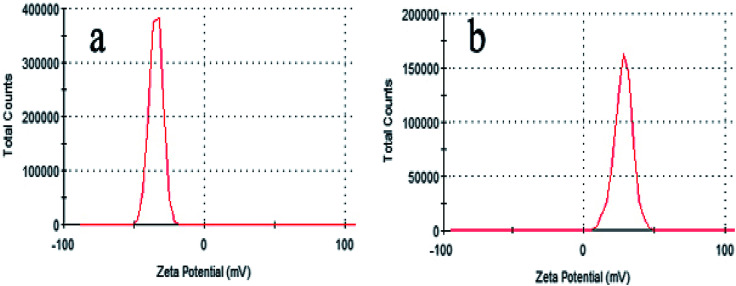
Zeta potentials of GO (a) and MoS_2_ (b) samples in water solution.

**Fig. 6:**
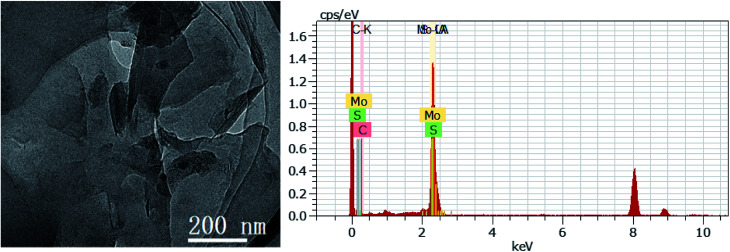
TEM and EDS images of GO/MoS_2_ composite.

According to the above-mentioned results, 3D GOMMZ composites were constructed using the layer-by-layer assembly process, as shown in Fig. 4. First, three-dimensional GO/MoS_2_ composites were spontaneously assembled by electrostatic attraction between GO and MoS_2_ nanoplates. From Fig. 5, we observe that the Zeta potential peak of GO is located at −34.3 mV (shown in Fig. 5a), whereas the peak of MoS_2_ is located at 28.3 mV (Fig. 5b), which indicated that GO and MoS_2_ nanosheets possessed negative charge and positive charge, respectively. When GO solution and MoS_2_ nanoplate solution were mixed, GO and MoS_2_ nanoplates were successfully assembled onto the three-dimensional GO/MoS_2_ structure due to electrostatic attraction. Fig. 6 shows TEM and EDS images of the 3D GO/MoS_2_ composite. It was very clear that MoS_2_ nanosheets can be deposited on GO sheets, and the composites were composed of C, Mo and S elements, which proved that MoS_2_ nanosheets were assembled on GO successfully.

Second, Mg-doped ZnO nanoparticles can be deposited on 3D GO/MoS_2_ sheets. When Zn^2+^ and Mg^2+^ are added, these positively charged metal ions are attracted by the 3D GO/MoS_2_ composites through the carboxyl groups on GO sheets or –NH_2_ groups on MoS_2_ nanoplates, and they are converted to ZnC_2_O_4_ and MgC_2_O_4_, respectively, after adding H_2_C_2_O_4_ solution. Eventually, zinc oxalate and magnesium oxalate were decomposed into ZnO and MgO nanoparticles after being annealed at 500 °C, and they are then supported on the 3D GO/MoS_2_ composites, which results in the formation of 3D GOMMZ composites.

**Fig. 7:**
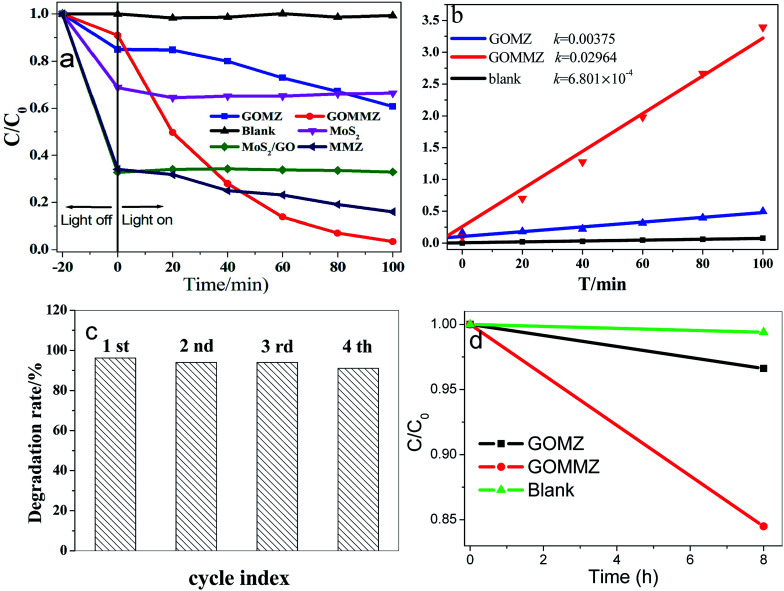
Photocatalytic degradation of different samples for RhB under visible light: (a) photocatalytic performance, (b) the degradation rate (*k*), (c) cyclic stability, and (d) photocatalytic activity under darkness.

Fig. 7 depicts the photocatalytic performances of different samples for RhB under visible light. Prior to visible-light exposure, the mixed solution is stirred in the dark for 20 min to reach the adsorption–desorption equilibrium. Clearly, MoS_2_, GO/MoS_2_ and MMZ samples display very strong adsorption abilities, but their photocatalytic activities are poor. The photocatalytic activity of the GOMZ sample is lower than that of the GOMMZ sample, and the degradation amount is only 30% within 100 min (shown in Fig. 7a). Moreover, the degradation amount of GOMMZ reaches nearly 100% within 100 min, and the degradation rate of GOMMZ is about 8 times as much as that of GMZ (Fig. 7b), implying that the photocatalytic activity of the GOMZ composite can be significantly improved by inserting MoS_2_ nanosheets on the GO sheets. To study the photocatalytic stability of GOMMZ, the sample is further studied by performing recycling experiments (as shown in Fig. 7c). The degradation rates for RhB reach nearly 100% within 100 min after four cycles, which indicates that GOMMZ has very high photocatalytic stability. Fig. 7d displays the photocatalytic activities of different samples under darkness. It is very clear that the GOMZ and GOMMZ samples possess photocatalytic activities to some extent even after irradiation is stopped, indicating that both GOMZ and GOMMZ can store some photoelectrons after irradiation by light, and they can release the electrons under darkness. Furthermore, we observe that the photocatalytic activity of GOMMZ is stronger than that of the GOMZ sample. These results show that MoS_2_ can improve the photocatalytic activity and photoenergy storage ability of the GOMZ sample.

**Fig. 8:**
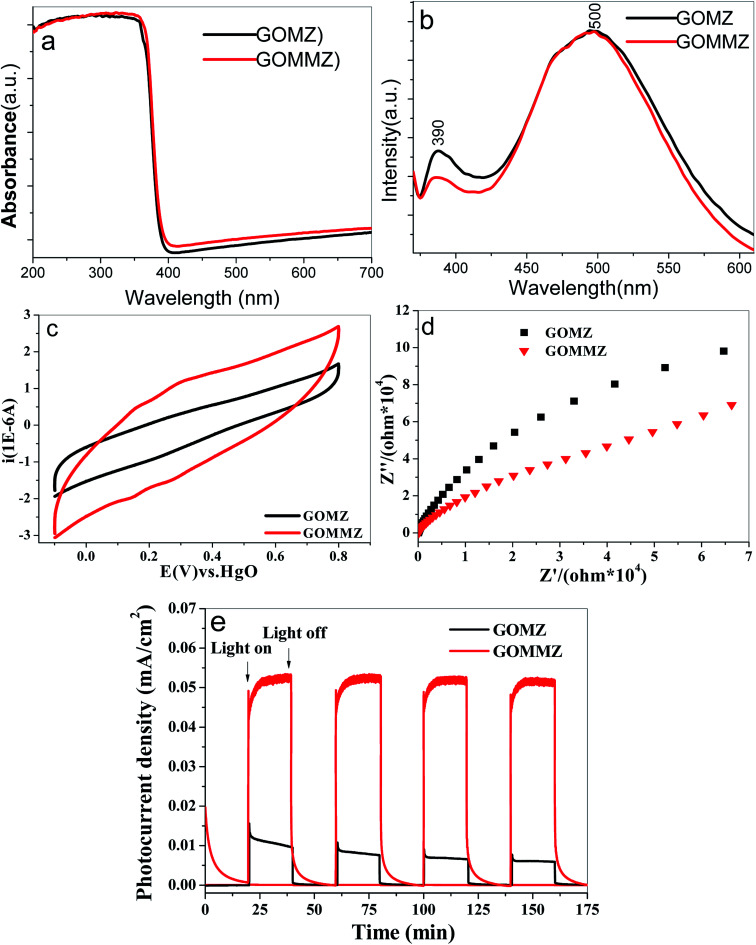
(a) UV-vis diffuse reflectance spectra, (b) room-temperature FL spectra, (c) CV curves, (d) Nyquist plots and (e) photocurrent spectra of GOMZ and GOMMZ samples.

The UV-vis diffuse reflectance spectra of GOMZ and GOMMZ are shown in Fig. 8a. A typical absorption in UV-light region with an edge at 390 nm can be seen in both GOMZ and GOMMZ, and their absorbances in visible light are similar. The absorption capacity of GOMMZ for visible light is stronger than that of the GOMZ composite, which indicates that MoS_2_ nanoplates broaden the corresponding spectrum range of the GOMZ composite. Fig. 8b shows the fluorescence spectra of GOMZ and GOMMZ at room temperature, which are obtained at the excitation wavelength of 340 nm. Both curves are similar and have two typical emission bands. The UV band at around 390 nm corresponds to the near-band-edge emission of ZnO due to the exciton recombination caused by an exciton collision process. The green emission band at about 500 nm is due to the singly ionized oxygen vacancy in the ZnO nanostructure.^43^ Normally, a weaker fluorescence peak represents lower recombination rate of photogenerated electron–hole pairs. Nevertheless, the quantum yield also has clear influence on the intensity of the fluorescence peak. Thus, the quantum yield of fluorescence is tested, and the photo-quantum yield of GOMZ is 2.5%, whereas that of GOMMZ increases to 3.5%. 3D GOMMZ shows lower intensity of the fluorescence peak and higher quantum yield than GOMZ, which indicates the lower recombination rate of the photogenerated electron–hole pairs. These results demonstrate that inserting MoS_2_ nanosheets can facilitate abundant photoexcitation, and it can also decrease the recombination rate of the electron–hole pairs.

Strong charge storage ability and small resistance are two key factors to improve the separation rate and transfer speed of photogenerated electron–hole pairs. Fig. 8c shows the CV curves of GOMZ and GOMMZ composites. We can observe that GOMMZ has larger integral area of the cyclic loop than GOMZ, which indicates that introducing MoS_2_ nanoplates can improve the specific capacitance of GOMZ composites. Based on the fact that the specific capacitance of electric double layer is proportional to the surface area of the electrode, we think that the enhancement in charge storage occurred due to the larger specific surface area and good electronic storage capability of MoS_2_ nanoplates. The Nyquist plots of GOMZ and GOMMZ are shown in Fig. 8d. It is clear that the reaction resistance of GOMMZ is smaller than that of GOMZ, indicating that the conductivity of GOMZ is improved by inserting MoS_2_ nanoplates onto GO sheets; this is due to the synergistic effect between GO and MoS_2_ because abundant interfaces between GO and MoS_2_ provide high-speed channels for electron migration. In addition, the photocurrents of different samples are measured under illumination and darkness (shown in Fig. 8e). We can clearly see that the photocurrent density of GOMMZ is five times higher than that of GOMZ under light source. When the light source is turned off, the photocurrent density of GOMZ drops to zero immediately, whereas the current of GOMMZ fades away gradually. These results suggest that the carrier density of GOMMZ is clearly higher than that of GOMZ under illumination, and GOMMZ has the ability to store charges.

These results show that the 3D GOMMZ composite possesses very high photocatalytic activity. The enhancement in photocatalytic activity is due to the effective synergistic effect between GO and MoS_2_ nanosheets. The 3D GO/MoS_2_ structure exhibits the following three functions: (i) MoS_2_ nanosheets improve the dispersion of GO, which increases the surface area and reduces the resistance, resulting in a decrease in the recombination of photogenerated electron–hole pairs. (ii) MoS_2_ nanosheets improve the utilization ratio for visible light as well as the optical quantum yield. (iii) MoS_2_ nanosheets can hinder the recombination of photogenerated electron–hole pairs because of their special structures and outstanding electrochemical properties.

**Fig. 9:**
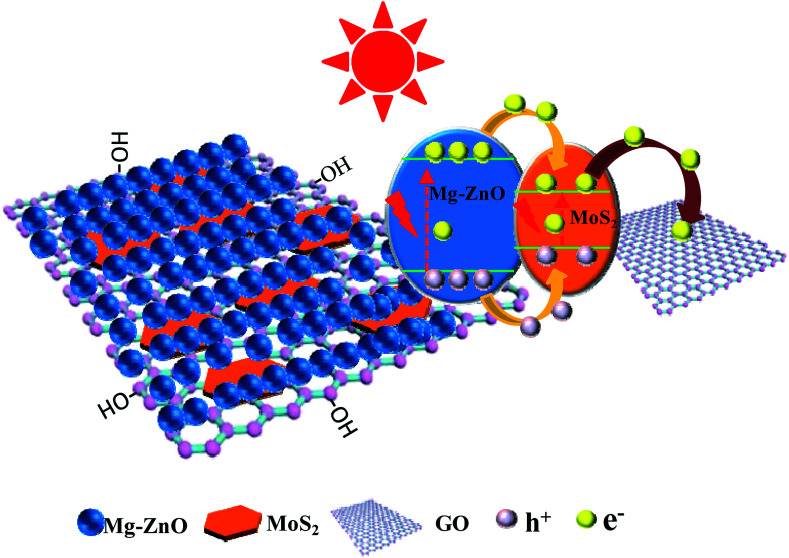
The scheme of electron transfer in ZnO, MoS_2_ and GO sheets.

The scheme of photocatalytic enhancement mechanism and electron transfer among ZnO, MoS_2_ and GO sheets is shown in Fig. 9. The band gaps of MoS_2_ nanoplates and ZnO were estimated to be 1.82 and 3.2 eV, respectively. The band edge of the conduction band (CB) was determined to be −0.08 V (*vs.* RHE) according to the empirical equation, and it was more positive than that of ZnO (*E*_CB_ = −0.31).^44−46^ When MoS_2_ and ZnO were integrated, the photogenerated electrons (e^−^) moved to the CB of the MoS_2_ nanoplate. Because the VB of the MoS_2_ nanoplate (1.74 eV) was lower than that of ZnO (2.89 eV), the photogenerated holes (h^+^) moved to the valence band (VB) of MoS_2_. Simultaneously, the work function value of GO could match well with that of the CB level of MoS_2_, leading to the photogenerated electrons in MoS_2_ nanoplates being transferred quickly to GO sheets, which retarded the recombination of photogenerated charges. Thus, the photocatalytic activity of GOMZ composite was improved by MoS_2_ nanoplates significantly. In addition, graphene can act as a sink for the photogenerated electrons, and it can store the photogenerated electrons in the large π–π network of graphene nanosheets. Moreover, MoS_2_ layer structure can also capture the photogenerated electrons, which changes the valency of Mo in MoS_2_ (Mo^4+^ to Mo^2+^ owing to obtaining photogenerated electrons under light irradiation, and Mo^2+^ can change to Mo^4+^ after losing light irradiation). Hence, we concluded that the GOMMZ composite possessed excellent photocatalytic activity and optical memory performance.

## 4 Conclusions

In summary, 3D GOMMZ composites have been successfully synthesized through electrostatic interaction, and the resultant 3D GOMMZ hybrids possess excellent photocatalytic activity and optical memory performance; the enhancement in photocatalytic activity is ascribed to the “synergistic effect” between GO and MoS_2_ nanosheets, and this increases the specific surface area and heightens the electronic conductivity, quantum yield and charge storage ability of the GOMZ composite. Moreover, the GOMMZ composite also shows optical memory ability. Our study provides an insight into the high performance of a photocatalyst by the synthesis of graphene oxide/other material composite carriers through electrostatic interaction methods.

## Conflicts of interest

There are no conflicts to declare.

## Supplementary Material
